# Investigation into the Prevalence of Cryptosporidium Infection in Calves among Small-Holder Dairy and Traditional Herds in Tanzania

**DOI:** 10.4061/2010/676451

**Published:** 2010-12-22

**Authors:** Emanuel S. Swai, Luuk Schoonman

**Affiliations:** ^1^Directorate of Veterinary Services, Veterinary Investigation Centre (VIC), P.O. Box 1068, Arusha 255, Tanzania; ^2^Department of Animal Production, Tanga Dairy Trust (TADAT), P.O. Box 1720, Tanga 255, Tanzania

## Abstract

A cross-sectional study was conducted to determine prevalence and risk factors of cryptosporidiosis in bovine from two contrasting production system in and around Tanga municipality between May 2003 and January 2004. The study populations comprised 117 calves aged ≤3 months, randomly selected from 44 smallholders dairy and traditional managed herds, respectively. Individual calf and herd-level information was collected using a structured questionnaire and feacal samples were screened for *Cryptosporidium* spp oocysts using the modified Ziehl-Neelsen method. Overall, 35% of the calves in the study were shedding *Cryptosporidium* spp oocysts, with at least one positive calf detected in 54.5% of herds. Independent risk factors for cryptosporidiosis were: age ≥1 to ≤2 months and level of cleanness of calf house floor categorized as dirty (*P* < .05). Similarly an increases risk of *Cryptosporidium* spp infection was found in calves from smallholder dairy units compared to traditional herds (*P* < .05). The finding highlights that *Cryptosporidium* spp is prevalent among calves in the area under study. The high prevalence of cryptosporidiosis detected in this study suggests that it may have a significant impact on livestock industry and that the close interaction between cattle and human may play a role in zoonotic transmission to humans.

## 1. Introduction

Cryptosporidiosis is a zoonosis caused by the apicomplexan intracellular, extracytoplasmic coccidian parasite of the genus *Cryptosporidium *that can infect a wide range of animals, including man [1, 2]. *Cryptosporidium hominis* (formerly *C. parvum* genotype 1) is human specific and maintained in human-to-human transmission cycles, while *C. parvum* (formerly *C. parvum* genotype 2) is maintained by a number of different animal reservoir host species including bovines [3]. *C. parvum *also causes disease in humans and is, therefore, a zoonosis transmitted from cattle to humans but in immunocompromised individuals such as children and patients with Acquired Immunodeficiency Syndrome (AIDS), the infection fulminates and might be life-threatening [4].

In cattle* Cryptosporidium* infections are transmitted by the faeco-oral route, and the disease is readily transmissible: oocysts persist for long periods in suitable environment [5], and low numbers of oocysts may produce infection in susceptible hosts [6]. Clinical illness and diarrhoea caused by *Cryptosporidium *spp. have been reported in several species of young animals including bovine calves as young as 4 days [7].

In Tanzania,* Cryptosporidium* spp. infections in human and livestock appear common [8–12]; however, systematic studies targeting young calves reared under different production system are patchy and not well documented. As a result information on disease prevalence, distribution, and impact on these future replacement stocks is inadequate and not precisely known.

This study investigated the prevalence of *Cryptosporidium* spp. infections amongst bovine calves in two contrasting cattle production systems of Tanga region, Tanzania. An attempt was made to investigate potential risk factors for infection in these calves that may point to methods by which infections may be controlled and/or transmission to humans limited.

## 2. Material and Methods

### 2.1. Study Area and Population

The study was conducted on both smallholder dairy herds (≤10 graded animals of all ages, breed, and sex and intensively managed) and traditional herds (≥30 indigenous cattle of all ages and sex and extensively managed) in and around Tanga municipality. Characteristics of the study area are described in detail elsewhere [13]. The type of animals kept under smallholder units includes taurine breed (Friesian, Ayrshire, Jersey, Simmental) and crosses of these breeds with bos indicus breeds (Tanzania shorthorn Zebu, Boran, and Sahiwal). The level of taurine blood varies from 50 to 85%. Traditional herds comprise mainly Tanzania shorthorn zebu (TSHZ). Only calves up to 3 months of age were sampled. Calves are known to be susceptible and suffer the severe form infections compared to other age group [5, 7].

### 2.2. Study Design and Herds Selection

This study was part of the broader cross-sectional study on zoonotic diseases and relative impact on public health conducted on the same farms between May 2003 and January 2004. During the cross-sectional study, 130 farms (105 smallholder dairies and 25 traditional herds) were selected randomly (using Epitable calculator of the Epi-info, version 6.04b) [14] from a sampling frame of 1,730 farms, comprising 12,500 animals that belonged to the Tanga dairy development programme and district livestock department databases. Recruitment of study calves was based on inclusion criteria of calves on the study farms with an age of less than three months as at 1st May 2003 through 1st January 2004. This selection criterion resulted in the recruitment of 28 smallholder dairy and 16 traditional herds comprising 117 calves. Consistent to other studies, cryptosporidiosis is considered one of the most common causes of neonatal diarrhea in cattle [7, 15]. All calves were sampled only once (cross-sectional) during the period of May 2003 and January 2004.

### 2.3. Questionnaire Design and Data Collection

Information about each herd and the calves kept was collected by means of a structured questionnaire, which was completed at all the selected herds on a single visit. The questionnaire was designed to comprise mostly closed ended (categorical) questions to ease data processing, minimize variation, and improve precision of responses [16]. The questionnaire was administered using the national Swahili dialect by a veterinary department staff member, who was trained in participatory research methodologies. Important herd-, calf- and area-level data recorded included calf location (urban, periurban, or rural), age determined from birth records, type of floor in the calf house (concrete, kraal/stone, wooden) as well as level of floor hygiene or cleanness subjectively assessed as highly, moderate, or mild dirty, and source of drinking water (tap, rain water, shallow well, river, pond). At the same time, information on diarrhoea and other ill health signs (coughing, dullness, ocular and nasal discharges) in calves was as well collected.

### 2.4. Sample Collection and Handling

During the visit to each herd, a fresh rectal faecal sample was collected from each calf into a sterile, airtight, 10 mL plastic tube. Collected faecal samples were labelled and transported in a cool-box to local laboratories in Tanga (at least within 6 hours of collection) prior to despatch in refrigerated containers to Sokoine University of Agriculture (SUA), Morogoro for analysis.

### 2.5. Laboratory Analysis of Fecal Samples

Presence of *Cryptosporidium* spp. oocysts in faeces samples was detected using the modified Ziehl-Neelsen staining technique as described by Clarke and McIntyre [17]. Briefly, faecal smears were prepared on a microscope slide, air dried at room temperature, and fixed with absolute alcohol (methanol) for 5 minutes. Fixed smears were stained with dilute carbol fuchsin (1 : 10) for 3–5 minutes and washed with tap water. Smears were decolourised using 3% acid alcohol (3% HCL in ethanol) for 10–15 minutes then counterstained with 0.5% malachite green solution for one minute. Smear slides were washed with tap water, air dried, and then examined under the microscope at x400 magnification. *Cryptosporidium* spp. oocysts appear as pink to red, spherical to ovoid bodies against a green to purple background. Samples were considered positive if at least one morphologically distinct *Cryptosporidium* spp. oocyst was observed. Intensities of the oocysts detected based at x400 magnification were expressed as 1+ (<5 oocyst per slide), 2+ (1 to 10 oocysts per field per view) and 3+ (>10 oocysts per field per view) [18].

### 2.6. Biostatistical Analysis

The data were stored using Microsoft Access and analyzed by using Epi-Info version 6.04d software (CDC, 1996) [14] statistical packages. Descriptive statistics like rate, frequency, and proportions were used. Risk factors thought to be associated with the prevalence of *Cryptosporidium* spp. oocyst shedding (age, location, production system, source of water, house flooring system, and level of cleanness) were evaluated using Chi-square analysis (Epi-Info version 6.04d software, CDC, 1996), with *P* < .05 as the indicative of significance level.

## 3. Results

### 3.1. Distribution of Risk Factors

Descriptions of herd-level and calf-level for the 117 calves sampled in the area under study are given in Table 1. Fifty seven percent of the sampled calves consisted of the indigenous breeds. Majority of the herds (63.6%) visited were smallholders dairy managed under exclusive zero grazing. Housing flooring systems was classed as made up of stones/kraal and were often found dirty (68%) at a time of visit. Approximately equal proportions of calves were sampled from the age category of one to two months. The mean age of the 117 calves investigated was 49 days (range 7–84 days), with 70 (60%) of the calves being rural residents. None of the examined calves were diarrheic or showing any obvious ill health signs. Further details are shown in Table 1.

### 3.2. Estimating Cryptosporidium spp Oocysts Infection Prevalence

**Figure 1:**
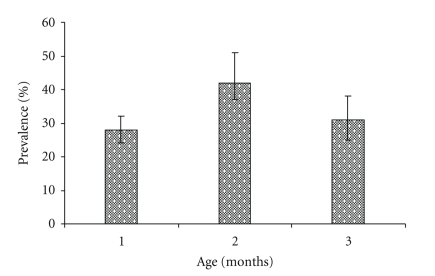
Age prevalence profile (±95%CI) of *Cryptosporidium *oocysts in the sampled calves in Tanga (May 2003–January 2004).

The overall individual calf- and herd-level cryptosporidiosis prevalence and their respective 95% Confidence intervals [CIs] are shown in Table 2. Calves in traditional herds had a significant lower prevalence compared to calves in smallholder herds (*P* < .05). The cryptosporidiosis prevalence profiles (with 95% CI for the exact binomial proportions) by age category are shown in Figure 1. Calves of ≥1 to ≤2 months of age had a higher prevalence than younger (≤1 month) and older calves (≤3 months) (*P* < .05). Based on oocysts detection intensities, 7.3% (4/41) of the *Cryptosporidium *spp positives were categorized as heavy, 9.7% (4/41) as moderate, and 83% (34/41) as mild infection.

### 3.3. Relationship between Variables Investigated and Cryptosporidium Oocyst Infection

One variable was significantly associated with variation in prevalence to *Cryptosporidium *spp infection in the univariable analysis. Calves were associated with a higher risk of exposure to *Cryptosporidium *spp infection if they slept/or stayed at dirty floors compared to those slept at moderate to clean floors (odd ratio [OR] = 1.82, 95%CI 1.15–3.02, *P* = .01 for dirty floors). At herd level, however, the type of flooring in the calf housing, either concrete or dirt flooring, did not influence the cryptosporidiosis herd prevalence. Also the water source, urban tap water as compared to water from shallow wells or surface water, was not associated with the cryptosporidiosis prevalence at herd level. None of the other investigated variables were associated with *Cryptosporidium *spp oocyst shedding.

## 4. Discussion

 In this study, there was evidence that, in and around Tanga municipality, calves are infected by *Cryptosporidium* spp, indicating that bovine cryptosporidiosis is endemic and locally widespread. The overall herd-level prevalence of 54.5% implies the presence of the disease in majority of the herds in the study area. Regarding production systems, 63% of herds in the traditional and 50% of the herds tested in smallholder dairying system had at least one *Cryptosporidium* spp. oocyst shedding calf per farm. The herd-level prevalence difference observed in the two production systems was not statistically significant (*P* > .05). This demonstrates the disease being important in both production systems. The recorded prevalence indicated, however, that certain herd-level factors, such as those involving close contact with unhygienic or dirty floor houses, were relatively high-risk in terms of *Cryptosporidium* spp infection. Some studies have shown that parasite oocysts are able to survive for extended periods in faeces and environment, and very low dose of viable oocysts can cause an infection [2]. The warm humid coastal environment of Tanga region may also favour the survival and spread of infective oocysts. 

The detected prevalence (35%) of infection in the calves was relatively higher than the 16.5% reported in diarrhoeic calves under 3 months of age in Morogoro, Tanzania [10], but generally lower than the prevalence observed in other studies of similar age range in Tanzania and elsewhere, for example, up to 62% [19] and 80% in calves in Britain [20]. 

The relevant data from North and East Africa, America, and from other environments similar to those found in Tanzania also indicate that bovine cryptosporidiosis is prevalent and widespread among calves populations. For example in other modified ZN screening-based investigations, prevalences of 86.7%; 38%; 18%; 17.1%, and 25% have been reported in Tunisia, Uganda, Kenya, Argentina, and Mexico, respectively [7, 21–24]. The apparent variability of prevalence between geographical localities and reports may reflect differences in the levels of calf management practices employed at farm level, housing-related factors (i.e., single housed calves, cleanness of the calf sleeping places), calf-related factors at a time of sampling (diarrhoeic versus nondiarrhoeic), nature of the study (cross-sectional versus prospective longitudinal studies), and fecal screening technique used [25–27]. 

Although modified ZN remains the widely used screening test for cryptosporidiosis, the test does have limitations [28–30]. Therefore, our prevalence estimates are likely to be at variance with the true prevalence in calves. A wider use of ZN under field conditions is constrained by the low sensitivity, time consuming (about 30 to 45 minutes), necessitating intensive training and experience to interpret the results. Resource constraints affecting logistics and laboratory capacity were the main reasons that prohibited utilization of tests of higher sensitivities in this study. However, consistent with the results of studies by [9, 19], the findings of this study indicate evidence of exposure to the *Cryptosporidium* spp. and suggest that there is a need for strict adherence to hygienic and good calf husbandry practice at farm level. It can also be recommended that other tests of higher sensitivities, that is, PCR, ELISA, should be employed in order to improve test sensitivity [27, 30–33].

Sex of the calf was not associated with *Cryptosporidium* spp. oocyst shedding in this study, and this is in agreement with the report of [34, 35]. The prevalence rate in middle age group (43%) showed statistically significant difference from young (27%) and older calves (31%) (*P* < .05). This finding was at variance with earlier reports [24, 36] where such age range was concluded to have no role in cryptosporidiosis epidemiology. Perhaps this assumption needs further investigation.

## 5. Conclusion

 In conclusion, overall animal and herd prevalence was high, suggesting that cryptosporidiosis is endemic and locally widespread disease. Calves were more likely to shed *Cryptosporidium *spp. positive oocysts if they were raised at dirty floor houses most likely due to the increased microenvironment for *Cryptosporidium *spp. oocyst survival and persistency. A further study prospective in nature, capturing seasonal variations to elucidate the magnitude of the disease (mortalities and reduced production), is desirable. Moreover, studies to understand the dynamics of transmission cycles and the genetic diversity of *Cryptosporidium *spp. on the farms, and to identify and if possible alter management practices that are risk factors for human infections, should be initiated and undertaken.

##  Conflict of Interests

The authors declare that they have no competing interests.

## Figures and Tables

**Table 1:** Characteristics of the 117 selected calves in terms of animal- and herd-level variables from Tanga (May 2003–January 2004).

Category	Levels	Frequency	Percentages
Herd-level factors (*n* = 44)			

Production system	Smallholders	28	63.6
	Traditional	16	36.4

Flooring system	Concrete	14	31.8
	Kraal/stones	28	63.6
	Wooden	2	4.5

Level of cleanness	Very dirty	9	20.4
	Slightly dirty	21	47.7
	Clean	14	31.8

Source of water	Tap water	22	50
	Shallow wells	4	9.09
	River, pond, rain	18	40.9

Area level factors (*n* = 117)			

Calf location	Urban	17	14.5
	Peri-urban	30	25.6
	Rural	70	59.9

Calf-factors (*n* = 117)			

Breed	Cross-bred	50	42.7
	Indigenous TSHZ	67	57.3

Age	≤1 month	37	42.7
	≥1 to ≤2 months	48	41.02
	≤3 months	32	27.3

Health status	Ill signs/diarrheic	0	0
	healthy	117	100

**Table 2:** Cryptosporidiosis prevalence (with exact ±95% confidence intervals) in calves up to 3 months of age by production system (May 2003–January 2004).

Production system	Herd level		Animal level	
	Number positives	Prevalence, % (±95%CI)	Number positives	Prevalence, % (±95%CI)
Traditional	10	63 (51.5–74.4)	15	22.4 (17.8–26.2)
Smallholder dairy	14	50 (40.7–59.3)	26	52 (45.1–58.9)
Overall	24	54.5 (45.6–60.4)	41	35 (30.9–39.1)

CI: lower and upper limits for 95 percent confidence interval of the prevalence.
